# Oncogenic *KRAS* Drives Metabolic Vulnerabilities by Directly Regulating Metabolic Enzymes in Cancer

**DOI:** 10.1055/s-0040-1712456

**Published:** 2020-07-08

**Authors:** Liyi Zhang

**Affiliations:** 1 State Key Laboratory of Oncology in South China, Collaborative Innovation Center for Cancer Medicine, Sun Yat-sen University Cancer Center, Guangzhou, Guangdong, People’s Republic of China

**Keywords:** cancer metabolism, metabolic reprogramming, aerobic glycolysis, *KRAS*, *KRAS4A* hexokinase 1

## Abstract

Metabolic reprogramming, such as enhanced aerobic glycolysis, allows cancer cells to maintain viability and promote proliferation. It is one of the major consequences of oncogenic mutations. *KRAS* is the most frequently mutated oncogene in human cancer. It is thought to be closely related to metabolic reprogramming. However, it is not clear whether it can participate in metabolic reprogramming by directly regulating metabolic enzymes. Additionally, the functional differences among the splice variants of *KRAS* have not been determined. In a study, recently published in *Nature* , Amendola et al reported a unique interaction between one of the *KRAS* splice variants ( *KRAS4A* ) and the major glycolytic enzyme (hexokinase 1) in cancer cells. Their findings indicated that a better understanding on the regulation of hexokinase 1 by *KRAS* may reveal novel therapeutic strategies.

## Introduction

Being able to obtain necessary nutrients from a nutrient-poor microenvironment, and using these nutrients to maintain viability and promote proliferation is a hallmark of cancer. 1 2 The alterations of metabolites caused by cancer-associated metabolic reprogramming have profound effects on cancer cell proliferation, metastasis, cancer stemness, and therapeutic resistance. 3 4 Existing studies reveal that targeting cancer metabolism may show potential therapeutic benefits for cancer treatment. 5 6

Cancer-associated metabolic reprogramming is one of the major consequences of oncogenic mutations. 7 8 *KRAS* , the most frequently mutated driver gene in cancer, is thought to be closely related to cancer-associated metabolic reprogramming. Previous studies have shown that oncogenic *KRAS* can increase glucose uptake and enhance the Warburg effect in cancer cells. 9 10 These metabolic effects of oncogenic *KRAS* have been explained by the upregulation of glucose transporters and glycolytic enzymes. 9 10 11 However, it is not clear whether it can participate in cancer-associated metabolic reprogramming by directly regulating metabolic enzymes.

KRAS4A and KRAS4B are the gene products of oncogenic *KRAS* and only differ in the C-terminal membrane targeting region. 12 Normal cells overexpressing either KRAS4A or KRAS4B can acquire the properties of cancer. 13 So far, the functional differences between them have not been determined. In a study, recently published in *Nature,* titled “KRAS4A directly regulates hexokinase 1”, Amendola et al 14 reported a direct and unique interaction between KRAS4A and hexokinase 1 (HK1) in cancer cells. They found that KRAS4A can directly bind to HK1 in mitochondria, thereby blocking the allosteric inhibition of HK1 which could lead to enhanced glycolytic flux in cancer cells.

In this study, the authors analyzed the proteins that directly interact with RAS proteins by coimmunoprecipitation of flag-tagged NRAS and mass spectrometry. They found that flag antibodies could, directly or indirectly, capture several proteins, including HK1, voltage-dependent anion channel (VDAC), and ADP/ATP translocase 1. Notably, VDAC could form a complex with HK1 on the outer mitochondrial membrane, 15 while simultaneously interacting with ADP/ATP translocase 1 on the inner mitochondrial membrane. 16 Furthermore, the authors validated the interaction between HK1 and NRAS by a series of coimmunoprecipitation assay. KRAS4A was found to be a subtype of RAS protein with the highest affinity for HK1. Additionally, the coimmunoprecipitation assays also revealed that HK1–KRAS4A interaction depended on GTP.

As we know, post-translational modifications such as palmitoylation and prenylation can drive differential transport of RAS proteins. Palmitoylation is unique to KRAS4A among the *KRAS* splice variants. The palmitoylation site of KRAS4A is on cysteine 180 in the C-terminal membrane targeting region. Palmitoylation of KRAS4A is necessary for its binding to plasma membrane 1. 12 However, C180S mutation often occurs in oncogenic *KRAS* . Therefore, the authors also explored whether the C180S mutation could affect KRAS4A palmitoylation. As the author speculated, the mutated KRAS4A could not be palmitoylated, which completely blocked all membrane association and thereby blocked the associations with HK1. Ultimately, this alteration led to diminished conversion of glucose into lactate.

From the data in this study, we can clearly deduce that HK1 meets all the requirements as an effector of KRAS4A because 1 HK1 could specifically bind to KRAS4A 2 ; HK1 and KRAS4A could combine in the GTP-bound form 3 ; and KRAS4A could change the activity of HK1. Many factors can lead to KRAS-induced metabolic reprogramming in cancer cells, including oncogenic KRAS which can increase the expression of metabolic-related transporters and enzymes. 9 10 This study sheds light on another possible mechanism: the direct regulation of HK1 activity for inducing cancer-associated metabolic reprogramming by KRAS. As HK1 has been shown to be the most important glycolytic enzyme that controls the glycolytic pathway, this suggests that the regulation of HK1 by KRAS4A will have a substantial effect in cancer initiation and progression. Because cancer cells must transfer glucose into the pentose phosphate pathway to maintain their rapid growth, blocking the direct regulation of HK1 by KRAS4A is a potentially effective method for treating cancer, especially for tumors with high oncogenic KRAS4A expression.
